# Mechanical insights into ribosomal progression overcoming RNA G-quadruplex from periodical translation suppression in cells

**DOI:** 10.1038/srep22719

**Published:** 2016-03-07

**Authors:** Tamaki Endoh, Naoki Sugimoto

**Affiliations:** 1 Frontier Institute for Biomolecular Engineering Research (FIBER), Konan University, 7-1-20 Minatojimaminamimachi, Kobe, 650-0047, Japan; 2 Graduate School of Frontiers of Innovative Research in Science and Technology (FIRST), Konan University, 7-1-20 Minatojimaminamimachi, Kobe, 650-0047, Japan

## Abstract

G-quadruplexes formed on DNA and RNA can be roadblocks to movement of polymerases and ribosome on template nucleotides. Although folding and unfolding processes of the G-quadruplexes are deliberately studied *in vitro*, how the mechanical and physical properties of the G-quadruplexes affect intracellular biological systems is still unclear. In this study, mRNAs with G-quadruplex forming sequences located either in the 5′ untranslated region (UTR) or in the open reading frame (ORF) were constructed to evaluate positional effects of the G-quadruplex on translation suppression in cells. Periodic fluctuation of translation suppression was observed at every three nucleotides within the ORF but not within the 5′ UTR. The results suggested that difference in motion of ribosome at the 5′ UTR and the ORF determined the ability of the G-quadruplex structure to act as a roadblock to translation in cells and provided mechanical insights into ribosomal progression to overcome the roadblock.

Guanine-rich (G-rich) sequences can form stable non-canonical structures called G-quadruplexes under physiological conditions^1^,^2^. Recent biological studies have revealed that both DNA and RNA G-quadruplexes form inside living cells^3^,^4^,^5^. Thus, this unique structure likely has a role in regulation of gene expression^6^,^7^.

DNA polymerase, RNA polymerase, reverse transcriptase, and ribosome, which show processive movement on template nucleotides, have fundamental functions for replication and expression of genomic information. Progression of all these proteins is known to be disturbed when G-quadruplex structure was formed on the template strand^6^,^8^,^9^,^10^,^11^. The proteins should unwind the G-quadruplex to continue their reactions. *In vitro* biophysical^12^,^13^,^14^ and mechanical^15^,^16^,^17^ analyses, and in silico simulations^18^,^19^,^20^ of folding and unfolding processes of the G-quadruplexes have suggested that the G-quadruplex unfolds through intermediate structures of partially folded quadruplex or G-triplex. Recent mechanical disruption studies done by Mao *et al*. demonstrated that the unfolding process of G-quadruplex differed depending on the positions, where the attracting force was applied, and resulted in changes in stability and unfolding kinetics of the G-quadruplex^21^,^22^. Based on the characteristic properties, progression of the polymerases and ribosome is expected to be differently affected by the G-quadruplex depending on their mechanisms to overcome the G-quadruplex region, although it is still challenging to correlate with intracellular biological reactions affected by the G-quadruplex.

G-quadruplexes located in both the 5′ untranslated region (UTR) and the open reading frame (ORF) of mRNAs reduce protein expression levels by acting as roadblocks to the ribosome^6^,^23^,^24^,^25^,^26^. From a mechanical viewpoint, only the small ribosomal subunit interacts with 5′ UTR and scans the region until a start codon is recognized, whereas the mature ribosome consisting of small and large subunits moves along the ORF by step-by-step translocation, which shows ratchet-like movement^27^,^28^,^29^,^30^. The mechanical driving forces of the small ribosomal sub unit and the mature ribosome are different^31^,^32^. In addition, since the mature ribosome progresses three nucleotides at an every step of translocation, there can be zero, one, or two nucleotides between the mRNA entry site of ribosome and the G-quadruplex when the mature ribosome approaches a G-quadruplex within the ORF. Thus, the ability of the G-quadruplex to block translation likely differs depending not only on whether it is located in the 5′ UTR or the ORF but also on its positions within ORF. However, little is known about positional effects of G-quadruplexes or of other RNA structures, such as pseudoknots and hairpins, that have suppressive effects on translation^33^,^34^.

In this study, we systematically evaluated the positional effect of a G-quadruplex forming sequence on the suppression of translation in cells. Reporter mRNAs, which form RNA G-quadruplex in the 5′ UTR or the ORF with stepwise displacement of their positions at single nucleotide levels, were designed. Based on a periodic fluctuation of translation suppression caused by RNA G-quadruplex within the ORF but not within the 5′ UTR, we propose mechanisms of single-step or two-step translocation to unwind the G-quadruplex during the translation elongation depending on the position of the G-quadruplex.

## Results

### Formation of G-quadruplex on reporter mRNA

**Figure 1:**
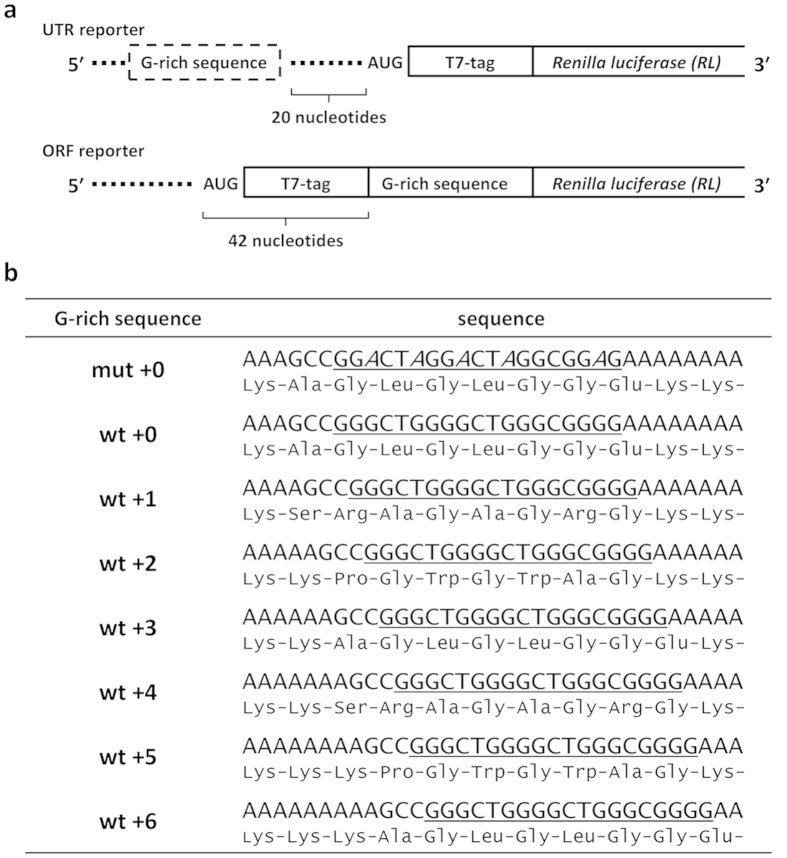
Design of reporter mRNAs. (**a**) A G-rich sequence derived from *E. coli eutE* gene or a variant was inserted into the 5′ UTR or the ORF of an mRNA encoding T7-tagged *Renilla* luciferase. (**b**) Sequences of G-rich sequence variants. Wild-type and mutant G-rich sequence regions are underlined. Positions of substitutions from guanine to adenine in the mutant sequence are in italics. Amino acid sequences encoded by the G-rich sequences within the ORF are shown under the nucleotide sequences.

Reporter mRNAs were constructed using a G-rich sequence derived from *E. coli eutE* gene; the reporter is based on a previously constructed reporter gene designed to express *Renilla* luciferase with a T7 tag at the N-terminus^26^. Mutant G-rich sequences with replacements of the guanine bases with adenine to prevent G-quadruplex formation were also constructed. Wild-type and mutant G-rich sequences were placed upstream or downstream of the start codon (Fig. 1a). Reporters containing wild-type G-rich sequences were created with stepwise displacement of the G-quadruplex forming sequence by a single nucleotide relative to the wt + 0 construct such that the G-quadruplex forming sequence was placed at seven different positions. This allowed us to evaluate the positional effect of the G-quadruplex with single-nucleotide resolution in both the 5′ UTR and the ORF (Fig. 1b).

**Figure 2:**
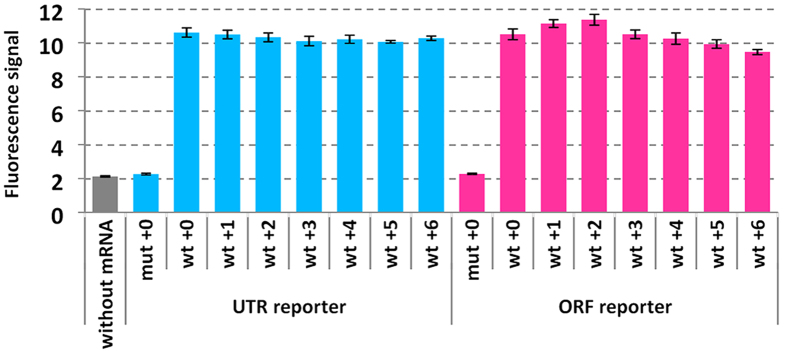
Analysis of NMM fluorescence in the presence of reporter mRNAs. Reporter mRNAs (500 nM) were mixed with NMM (5 μM) in a buffer containing 30 mM HEPES-KOH (pH 6.8), 100 mM KCl, 0.1% DMSO, and 0.01% Tween 20. Florescence intensities were measured after incubation at 37 °C for 30 min using 400 nm excitation and 615 nm emission. mRNAs were purified and refolded by heating at 70 °C for 5 min and cooling to 25 °C at 1 °C min^−1^ before addition of NMM.

The reporter mRNAs were transcribed and formation of G-quadruplex on the mRNAs was evaluated by analysis of the fluorescence signal of *N*-methyl mesoporphyrin (NMM). NMM fluorescence is enhanced upon binding to a G-quadruplex^35^,^36^. Fluorescence signals of NMM with reporter mRNAs containing the wild-type G-rich sequence were higher than those without mRNA and with mRNAs containing mutant sequence irrespective of position of the G-rich sequence (Fig. 2). The signal intensities among the wild-type mRNAs were almost the same in the presence of 10-fold excess of NMM. In our previous study, a short RNA oligonucleotide corresponding to the wild-type G-rich sequence formed parallel G-quadruplex^26^. The flanking sequences of the wild-type G-rich sequence that may affect G-quadruplex stability and topology^37^,^38^ are designed to be all the same with 5′ GCC and 3′ AA. Thus, it was suggested that the wild-type G-rich sequences in the reporter mRNAs uniformly formed parallel G-quadruplex.

### Intracellular translation suppression caused by G-quadruplex

**Figure 3:**
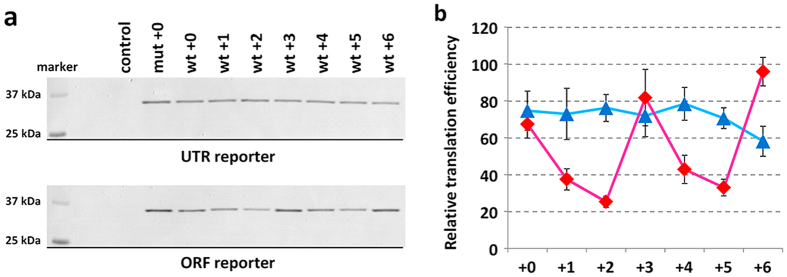
Translation suppression in MCF7 cells caused by RNA G-quadruplexes located in the 5′ UTR and ORF. (**a**) Translated products from reporter mRNAs detected by western blotting. (**b**) Translation efficiencies of 5′ UTR reporter (blue) and ORF reporter (red) mRNAs. The values were calculated by dividing relative R/F protein ratio by relative R/F mRNA ratio (see Figs S2b and S3, in the Supporting Information).

Effects of the G-quadruplexes on protein expression were evaluated in a cell-based assay. Plasmids encoding the reporter mRNAs under control of Cytomegalovirus (CMV) promoter were transfected into MCF7 human breast carcinoma cells. Plasmid vector (pSV40-FLuc), which expresses firefly luciferase under Simian vacuolating virus 40 (SV40) enhancer and early promoter, was co-transfected to normalize transfection efficiency of the experimental samples. Cells were lysed 24 hours after transfection and expression levels of firefly luciferase were evaluated by luminescence signals (see Fig. S1a, in the Supporting Information). Expression levels of *Renilla* luciferase in the same lysate were evaluated by western blotting using an anti-T7 tag antibody (Fig. 3a). The signal intensities of the western blotting were normalized by luminescence signals of firefly luciferase to obtain relative expression levels of *Renilla* luciferase (R/F protein ratio) (see Fig. S1b, in the Supporting Information). The R/F protein ratios in cells that expressed the reporter mRNAs with the wild-type G-rich sequence both in the 5′ UTR and in the ORF were lower than in cells that expressed the mRNAs with the mutant G-rich sequence at cognate locations. The R/F protein ratios were periodically changed depending on the position of wild-type G-rich sequence within the ORF while those were the same within experimental error irrespective of the position in the 5′ UTR.

Previous studies have suggested that the transcription of G-rich sequence induced formation of a DNA/RNA hybrid G-quadruplex that suppresses transcription levles^39^,^40^,^41^. To accurately investigate the positional effect of G-quadruplex on translation reaction, levels of mRNA transcripts should be considered. Transcription levels of *Renilla* luciferase mRNA relative to those of firefly luciferase mRNA (R/F mRNA ratio) were evaluated by real-time PCR (see Fig. S2, in the Supporting Information). Moderately reduced R/F mRNA ratios in the cells that transcribed mRNAs with wild-type G-rich sequences comparing to the cells that transcribed mRNA having mutant G-rich sequence suggest that the transcription of the wild-type G-rich sequence induced formation of the DNA/RNA hybrid G-quadruplex^39^,^40^,^41^ in cells. However, there was no positional effect of the wild-type G-rich sequence on the R/F mRNA ratio irrespective of the 5′ UTR or the ORF.

Intracellular translation efficiencies of the mRNAs with the wild-type G-rich sequence relative to the mRNA with the mutant G-rich sequence were evaluated from R/F protein ratio and R/F mRNA ratio (Fig. 3b). The translation efficiencies of the wild-type G-rich sequence in all positions in the 5′ UTR and the ORF were lower than that of the mutant sequence. The results clearly showed periodic fluctuation of translation suppression at every three nucleotides within the ORF but not within the 5′ UTR, although the translation suppression caused by the ORF wt + 6 was slight and probably within error of the mutant reporter.

### General property of the translation suppression in various cell lines

**Figure 4:**
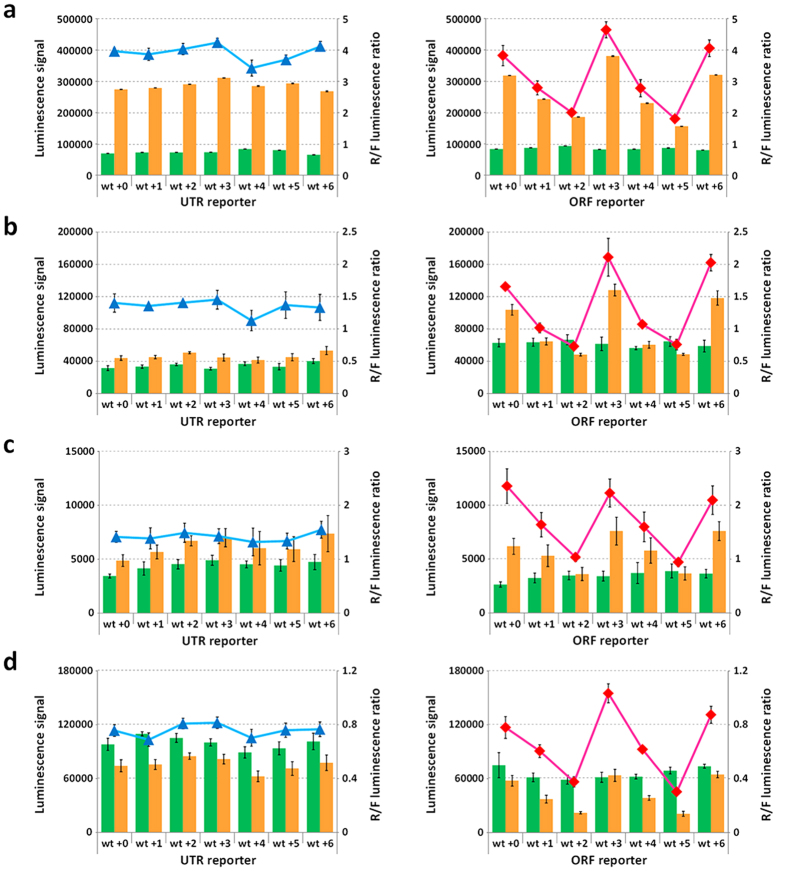
Dual luciferase assay in cell lysates from (**a**) MCF7, (**b**) Flp-Iin 293, (**c**) HeLa, and (**d**) HepG2 cells transfected with reporter and control plasmids. Luminescence signals of firefly (green) and *Renilla* (orange) luciferases (left axes) are plotted in bar graphs; R/F luminescence ratios of 5′ UTR reporter (blue) and ORF reporter (red) (right axes) are plotted as data points. Values are means ± S.D. obtained from 4 or 5 wells.

To confirm whether the periodic fluctuation of translation suppression can be observed in different cell lines, we quantitatively analyzed protein expression levels of *Renilla* luciferase by using dual luciferase assay. We assumed that the differences in amino acid sequences at the G-rich sequence region do not affect the relative activity of *Renilla* luciferase. This assumption was justified since the luminescence signals of *Renilla* luciferase relative to firefly luciferase (R/F luminescence ratio) in MCF7 cells (Fig. 4a) almost corresponded to the R/F protein ratio (Fig. S1b, in the Supporting Information). The periodic fluctuations of R/F luminescence ratios within the ORF but not within the 5′ UTR were also observed in human embryonic kidney (Flp-In 293, Invitrogen), cervix epithelioid carcinoma (HeLa), and hepatocellular carcinoma (HepG2) cells (Fig. 4b–d). In addition, the fluctuation of R/F luminescence ratios was also observed when the G-rich sequence variants were located at 792-nucleotides downstream of the start codon (Fig. S3, in the Supporting Information).

ORF reporter mRNAs having G-rich sequence derived from human E4F transcription factor 1 (*E4F1*) gene, which is encoding a key regulator protein of p53 tumor suppressor protein^42^,^43^, at different positions were also constructed (Fig. S4a, in the Supporting Information). The periodic fluctuations of R/F luminescence ratios at every three nucleotides were observed in all cells tested (Fig. S4b–e, in the Supporting Information). From these results, it was suggested that the positional effect of the G-quadruplex on translation suppression is general property caused by the structure that depends on mechanical and physical properties of the G-quadruplex and the translational machinery.

## Discussion

**Figure 5:**
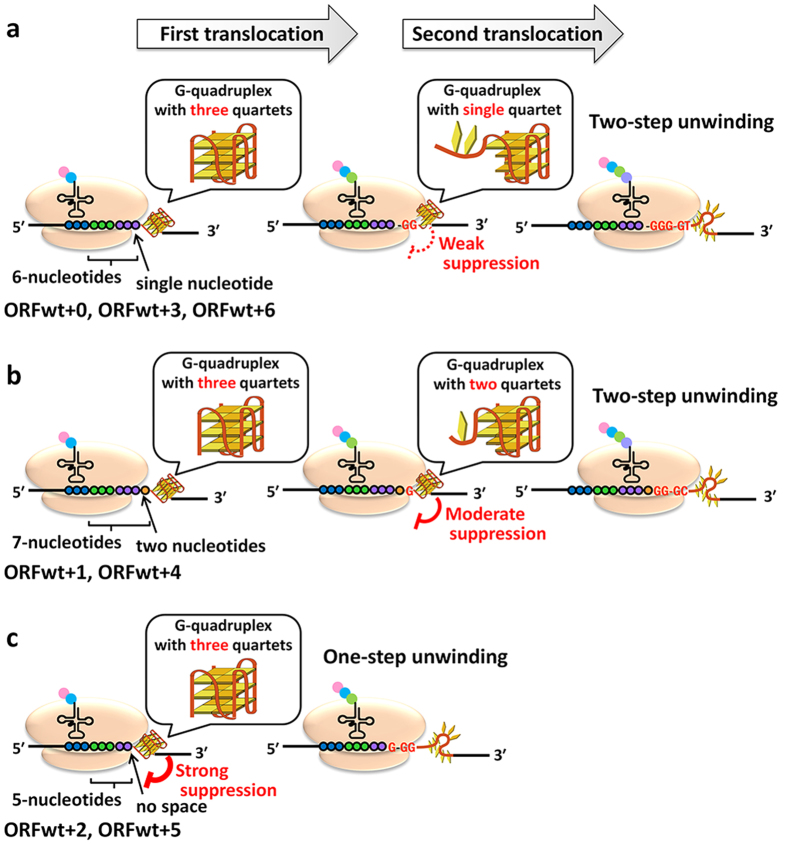
Schematic illustration of effects of RNA G-quadruplexes located at (**a**) wt + 0, wt + 3, and wt + 6, (**b**) wt + 1 and wt + 4, and (**c**) wt + 2 and wt + 5 positions of ORF on translation.

Previous studies demonstrated that, within the 5′ UTR of an mRNA, the extent of translational inhibition depended on stability of the G-quadruplex and its relative position to a site for ribosomal binding^44^,^45^. The G-quadruplex positions investigated in the previous studies were dispersed broadly across the 5′ UTR. In contrast to the studies, here, we evaluated effects of displacement of the G-quadruplex within a range of seven adjacent nucleotides with considering levels of mRNA transcripts. We observed no differences in translation efficiency from the UTR reporter mRNAs with the wild-type G-quadruplex sequences at positions 0 (UTR wt + 0) to 6 (UTR wt + 6). The results suggest that small displacement on 5′ UTR do not affect the translation efficiency. In contrast, the translation efficiency fluctuated significantly when the G-quadruplex forming sequences derived from both *E.coli* and human genes were altered in single-nucleotide steps in the ORF; translation efficiency oscillated with a three-nucleotide period when the G-quadruplex was located in the ORF. The oscillation of the translation suppression was irrespective of the G-rich sequences and their locations within ORF. In our previous *in vitro* study, translation elongation was halted when there were 5, 6 or 7 nucleotides between a codon in the A-site and the first guanine of the G-rich sequence^35^. A similar phenomenon was observed in mammalian cells^46^. Various *in vitro* mechanical studies and in silico simulations suggest that the G-quadruplex unfolds through intermediate of partially unfolded G-quadruplex or G-triplex, while there are possibilities to form multiple intermediate states^15^,^16^,^17^,^18^,^19^,^20^,^47^,^48^. Here, based on these previous reports and results in this study, we hypothesize that the ribosome unwinds the G-quaduplex in two-step translocation through the intermediate of partially unwound G-quadruplex or G-triplex, and propose the model shown in Fig. 5. When the translation elongation is halted before the G-quadruplex formed on ORF wt + 0, wt + 3, and wt + 6 mRNAs, there is a single-nucleotide space between the mRNA entry site of ribosome and the downstream G-quadruplex. In this case, the ribosome overcomes the G-quadruplex region through an intermediate, in which two guanine bases in the quartets are pulled into the ribosome and a single G-quartet is remained (Fig. 5a, middle part). For ORF wt + 1, and wt + 4 mRNAs, the two-step translocation goes through the intermediate, in which a single guanine is pulled into the ribosome and two G-quartets are present, because there is two-nucleotides spacer between the ribosome and the G-quadruplex before the first translocation (Fig. 5b, middle part). The intermediate should be more stable for the ORF wt + 1 and wt + 4 mRNAs than for the ORF wt + 0, wt + 3, and wt + 6 mRNAs, and this correlates with the lower translation efficiencies of ORF wt + 1 and wt + 4 mRNAs compared to those of ORF wt + 0, wt + 3 and wt + 6 mRNAs. The translation efficiency of the ORF wt + 2 and wt + 5 mRNAs were expected to be the lowest as the ribosome should unwind the G-quadruplex by one-step translocation since all quartets of the G-quadruplex are “in frame” with the first translocation (Fig. 5c). This should enforce ribosome the highest energy to overcome the G-quadruplex region.

Recent studies have suggested that a stable RNA structure within an ORF causes arrhythmic translation by inducing a translational halt^49^,^50^,^51^,^52^,^53^. Stalling of a ribosome can result in internal translation initiation^52^, ribosomal frameshift^53^, no-go mRNA decay^49^, or altered co-translational folding of nascent protein^51^. The results detailed here reveal that the same G-quadruplex forming sequence suppressed translation in both the 5′ UTR or the ORF. More interestingly, the extent of translation suppression due to the G-quadruplex in the ORF depended on the relative position and distance between ribosome and the G-quadruplex at single nucleotide level. We expect that the thermodynamic stabilities of G-quadruplexes formed were the same independent of location because the G-quadruplex forming and flanking sequences were identical. Thus, the intracellular periodic fluctuation of the translation suppression observed when the G-quadruplex is formed in the ORF seems due to differences in motion of ribosome to unwind a G-quadruplex. Triplet periodicity within the ORF region is also observed in natural biological phenomena such as siRNA efficiency and mRNA stabilities^54^,^55^,^56^. Our study sheds light on these intriguing biological fluctuations from mechanics of step-by-step translocation and unfolding of RNA structures by the ribosome.

## Methods

### Construction of plasmid vectors

Plasmid vectors encoding *Renilla* luciferase with a G-quadruplex forming sequence in various positions in the 5′ UTR or the ORF under control of a cytomegalovirus promoter were constructed based on the previously described pCMV-TnT-T7-RL reporter vector^26^. Plasmid vectors for ORF wt + 0 and ORF mut + 0 were previously constructed^26^. DNA fragments having G-rich sequence derived from *E. coli eutE* and human *E4F1* genes at different positions were prepared by annealing sense and antisense oligonucleotides shown in Tables S1 and S2, in the Supporting Information. The DNA fragments were cloned into NheI and XhoI sites or EcoRI and SalI sites of pCMV-TnT-T7-RL to construct 5′ UTR or ORF reporter vectors, respectively. All sequences inserted into the plasmid were confirmed. The coding sequence of AcGFP was excised from pAcGFP-C1 (Clontech) using NheI and XhoI, and was cloned into same restriction enzyme sites of ORF reporter vector to construct reporter genes having G-rich sequence at the middle of ORF.

### Preparation of mRNAs

DNA templates for *in vitro* transcription of reporter mRNAs were prepared by PCR amplification from the plasmid vectors using T7 promoter primer, 5’-CTTAATACGACTCACTATAGG-3’, and the antisense primer, 5′–CACTGCATTCTAGTTGTGGTTTG–3′. Reporter mRNAs were transcribed using ScriptMAX Thermo T7 Transcription Kit (Toyobo). Transcripts were purified using the RNeasy Mini Kit (Qiagen) and desalted using Centrisep Spin columns (Princeton).

### Fluorescence measurements

mRNAs were refolded in a buffer containing 30 mM HEPES-KOH (pH 6.8) and 100 mM KCl by heating to 90 °C followed by cooling at a rate of 1 °C min^–1^. NMM (5 μM) was mixed with the refolded mRNAs (500 nM) in a buffer containing 30 mM HEPES-KOH (pH 6.8), 100 mM KCl, and 0.1% DMSO. Fluorescence intensity of NMM was measured at 37 °C using a microwell plate reader (Infinite 200 Pro; Tecan) with 400 nm excitation and 615 nm emission.

### Transfection

MCF7, Flp-In 293, HeLa, and HepG2 cells were cultured in EMEM, DMEM, DMEM, and EMEM, respectively, supplemented with 10% fetal bovine serum and antibiotics (100 U/mL penicillin and 100 μg/mL streptomycin), and maintained at 37 °C under 5% CO_2_. Cells were plated on collagen-coated 96- or 24-well plates and incubated overnight. Reporter plasmids (50 or 250 ng) and control plasmid, pSV40-FLuc (5 or 25 ng) were co-transfected into cells cultured on 96- or 24- well plates, respectively, with 0.3 or 1.5 μL of X-tremeGENE 9 (Roche), respectively, according to the manufacturer’s protocol.

### Western blotting

Cell lysates in a Passive Lysis Buffer (Promega) were separated on a 12.5% e-PAGEL (Atto). Proteins in the gel were transferred to PVDF membrane using iBlot Dry Blotting System (Thermo Fisher Scientific), and *Renilla* luciferase was detected by an anti-T7 tag mouse monoclonal antibody (Novagen) and anti-mouse IgG antibody conjugated with alkaline phosphatase (Promega) using iBind Western System (Thermo Fisher Scientific). Signal intensities on the membrane were calculated using Multi Gauge software (Fuji Film) after the membrane was scanned as a tif image.

### Real-time PCR

Total RNAs were obtained using the RNeasy Mini Kit (Qiagen) 24 h after transfection and treated with DNase. mRNAs were converted to cDNAs by PrimeScript RT-PCR Kit (Takara) using oligo-dT primer. Levels of firefly and *Renilla* luciferase mRNAs were evaluated by StepOnePlus Real-Time PCR System (Thermo Fisher Scientific) using primers sets shown in Table S3, in the Supporting Information.

### Dual luciferase assay

Cells were lysed in a Passive Lysis Buffer at 24 h after transfection. Aliquots of the lysates were transferred to wells of a 384-well white plate. Luminescence signals from firefly and *Renilla* luciferases in the lysate were measured using the Dual-Glo Luciferase Assay System (Promega) in a microwell plate reader (Varioskan Flash; Thermo Scientific). Luminescence signal of *Renilla* luciferase relative to firefly luciferase (R/F luminescence ratio) was calculated after subtraction of luminescence signals obtained from Passive Lysis Buffer only.

## Additional Information

**How to cite this article**: Endoh, T. and Sugimoto, N. Mechanical insights into ribosomal progression overcoming RNA G-quadruplex from periodical translation suppression in cells. *Sci. Rep.* **6**, 22719; doi: 10.1038/srep22719 (2016).

## Supplementary Material

Supplementary Information
