# Supplementary material for: Mechanical insights into ribosomal progression overcoming RNA G-quadruplex from periodical translation suppression in cells
Source: Sci Rep. 2016 Mar 7;6:22719. doi: 10.1038/srep22719 (PMC4780275; doi:10.1038/srep22719)
Supplement: Supplementary Information [file srep22719-s1.doc]

**Supporting Information**

Mechanical insights into ribosomal progression overcoming RNA G-quadruplex from periodical translation suppression in cells

Tamaki Endoh^1^ and Naoki Sugimoto^1,2 *^

^1^Frontier Institute for Biomolecular Engineering Research (FIBER), Konan University, 7-1-20 Minatojimaminamimachi, Kobe, 650-0047, Japan

^2^Faculty of Frontiers of Innovative Research in Science and Technology (FIRST), Konan University, 7-1-20 Minatojimaminamimachi, Kobe, 650-0047, Japan

E-mail: [sugimoto@konan-u.ac.jp](mailto:sugimoto@konan-u.ac.jp) ;

Fax: +81-78-303-1495; Tel: +81-78-303-1457

Table S1. DNA oligonucleotides for construction of reporter plasmids

| Oligonucleotides for inserting G-rich sequence variants derived from *E. coli eutE* gene into 5′ UTR or ORF | | | |
| --- | --- | --- | --- |
| UTR reporter | | DNA sequence |  |
| mut +0 | sense  antisense | CTAGCATTTAGGTGACAAAGCCGGACTAGGACTAGGCGGAGAAAAAAAAC  TCGAGTTTTTTTTCTCCGCCTAGTCCTAGTCCGGCTTTGTCACCTAAATG |  |
| wt +0 | sense  antisense | CTAGCATTTAGGTGACAAAGCCGGGCTGGGGCTGGGCGGGGAAAAAAAAC  TCGAGTTTTTTTTCCCCGCCCAGCCCCAGCCCGGCTTTGTCACCTAAATG |  |
| wt +1 | sense  antisense | CTAGCATTTAGGTGACAAAAGCCGGGCTGGGGCTGGGCGGGGAAAAAAAC  TCGAGTTTTTTTCCCCGCCCAGCCCCAGCCCGGCTTTTGTCACCTAAATG |  |
| wt +2 | sense  antisense | CTAGCATTTAGGTGACAAAAAGCCGGGCTGGGGCTGGGCGGGGAAAAAAC  TCGAGTTTTTTCCCCGCCCAGCCCCAGCCCGGCTTTTTGTCACCTAAATG |  |
| wt +3 | sense  antisense | CTAGCATTTAGGTGACAAAAAAGCCGGGCTGGGGCTGGGCGGGGAAAAAC  TCGAGTTTTTCCCCGCCCAGCCCCAGCCCGGCTTTTTTGTCACCTAAATG |  |
| wt +4 | sense  antisense | CTAGCATTTAGGTGACAAAAAAAGCCGGGCTGGGGCTGGGCGGGGAAAAC  TCGAGTTTTCCCCGCCCAGCCCCAGCCCGGCTTTTTTTGTCACCTAAATG |  |
| wt +5 | sense  antisense | CTAGCATTTAGGTGACAAAAAAAAGCCGGGCTGGGGCTGGGCGGGGAAAC  TCGAGTTTCCCCGCCCAGCCCCAGCCCGGCTTTTTTTTGTCACCTAAATG |  |
| wt +6 | sense  antisense | CTAGCATTTAGGTGACAAAAAAAAAGCCGGGCTGGGGCTGGGCGGGGAAC  TCGAGTTCCCCGCCCAGCCCCAGCCCGGCTTTTTTTTTGTCACCTAAATG |  |
| ORF reporter ^(a^ | |  |  |
| wt +1 | sense  antisense | AATTCAAAAGCCGGGCTGGGGCTGGGCGGGGAAAAAAAG  TCGACTTTTTTTCCCCGCCCAGCCCCAGCCCGGCTTTTG |  |
| wt +2 | sense  antisense | AATTCAAAAAGCCGGGCTGGGGCTGGGCGGGGAAAAAAG  TCGACTTTTTTCCCCGCCCAGCCCCAGCCCGGCTTTTTG |  |
| wt +3 | sense  antisense | AATTCAAAAAAGCCGGGCTGGGGCTGGGCGGGGAAAAAG  TCGACTTTTTCCCCGCCCAGCCCCAGCCCGGCTTTTTTG |  |
| wt +4 | sense  antisense | AATTCAAAAAAAGCCGGGCTGGGGCTGGGCGGGGAAAAG  TCGACTTTTCCCCGCCCAGCCCCAGCCCGGCTTTTTTTG |  |
| wt +5 | sense  antisense | AATTCAAAAAAAAGCCGGGCTGGGGCTGGGCGGGGAAAG  TCGACTTTCCCCGCCCAGCCCCAGCCCGGCTTTTTTTTG |  |
| wt +6 | sense  antisense | AATTCAAAAAAAAAGCCGGGCTGGGGCTGGGCGGGGAAG  TCGACTTCCCCGCCCAGCCCCAGCCCGGCTTTTTTTTTG |  |
| 1. ORF wt+0 and ORF mut+0 were previously constructed.^1^ | | |  |

1. Endoh, T., Kawasaki, Y. & Sugimoto, N. Suppression of Gene Expression by G-quadruplexes in Open Reading Frames Depends on G-Quadruplex Stability*. Angew. Chem. Int. E*d**.** 52, 5522-5526 (2013).

Table S2. DNA oligonucleotides for construction of reporter plasmids

| Oligonucleotides for inserting G-rich sequence variants derived from human *E4F1* gene into ORF | | |
| --- | --- | --- |
| E4F1 ORF reporter | |  |
| wt +0 | sense  antisense | AATTCAAAGCCGGGCGGGAAGCGGGCGAGGGTGCAAAAAAAG  TCGACTTTTTTTGCACCCTCGCCCGCTTCCCGCCCGGCTTTG |
| wt +1 | sense  antisense | AATTCAAAAGCCGGGCGGGAAGCGGGCGAGGGTGCAAAAAAG  TCGACTTTTTTGCACCCTCGCCCGCTTCCCGCCCGGCTTTTG |
| wt +2 | sense  antisense | AATTCAAAAAGCCGGGCGGGAAGCGGGCGAGGGTGCAAAAAG  TCGACTTTTTGCACCCTCGCCCGCTTCCCGCCCGGCTTTTTG |
| wt +3 | sense  antisense | AATTCAAAAAAGCCGGGCGGGAAGCGGGCGAGGGTGCAAAAG  TCGACTTTTGCACCCTCGCCCGCTTCCCGCCCGGCTTTTTTG |
| wt +4 | sense  antisense | AATTCAAAAAAAGCCGGGCGGGAAGCGGGCGAGGGTGCAAAG  TCGACTTTGCACCCTCGCCCGCTTCCCGCCCGGCTTTTTTTG |
| wt +5 | sense  antisense | AATTCAAAAAAAAGCCGGGCGGGAAGCGGGCGAGGGTGCAAG  TCGACTTGCACCCTCGCCCGCTTCCCGCCCGGCTTTTTTTTG |
| wt +6 | sense  antisense | AATTCAAAAAAAAAGCCGGGCGGGAAGCGGGCGAGGGTGCAG  TCGACTGCACCCTCGCCCGCTTCCCGCCCGGCTTTTTTTTTG |
|  | | |

Table S3. DNA primers for RT-PCR

| Target gene | | DNA sequence |
| --- | --- | --- |
| Firefly luciferase | sense  antisense | CAACACCCCAACATCTTCGAC  TCGCGGTTGTTACTTGACTG |
| *Renilla* luciferase | sense  antisense | TTACATGGTAACGCGGCCTC  GAAATGCCAAACAAGCACCC |


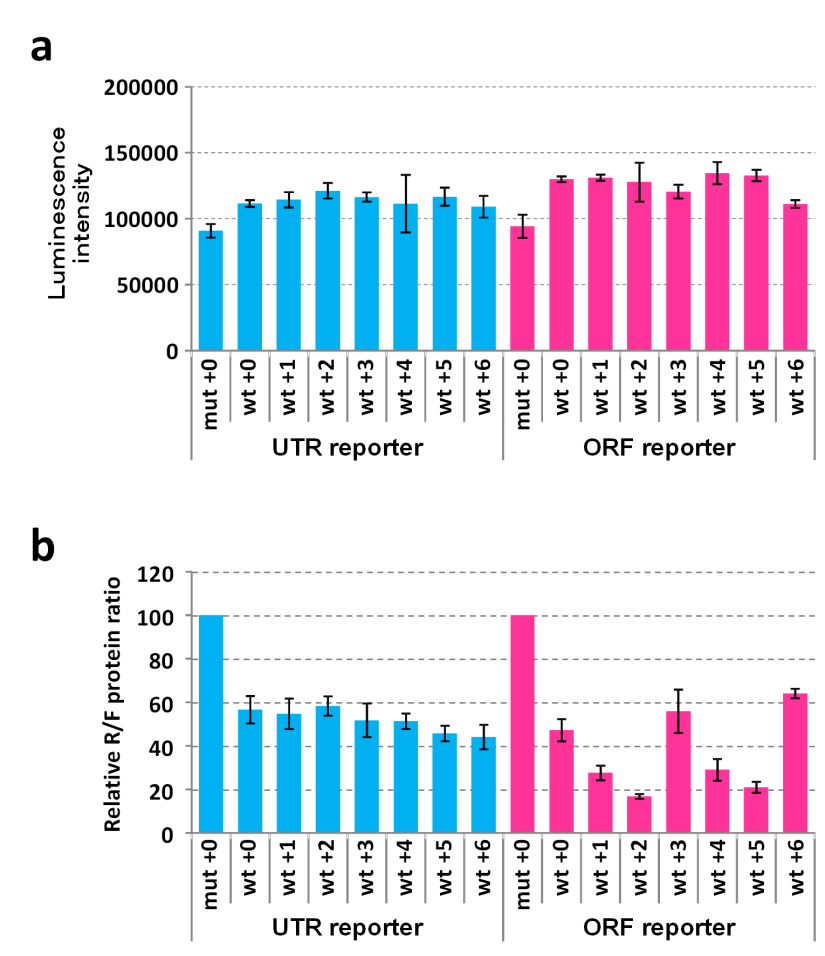


**Figure S1.** a) Luminescence intensities of firefly luciferase in cell lysate. MCF7 cells in 24-well plate were transfected with plasmid vectors and lysed after 24 hours. b) R/F protein ratios of indicated mRNAs relative to that of mRNA with mutant G-rich sequence. Signal intensities of western blotting in Figure 2a were normalized by the luminescence singals of firefly luciferase in (a). Values are means ± S.D. of triplicate experiments.

**
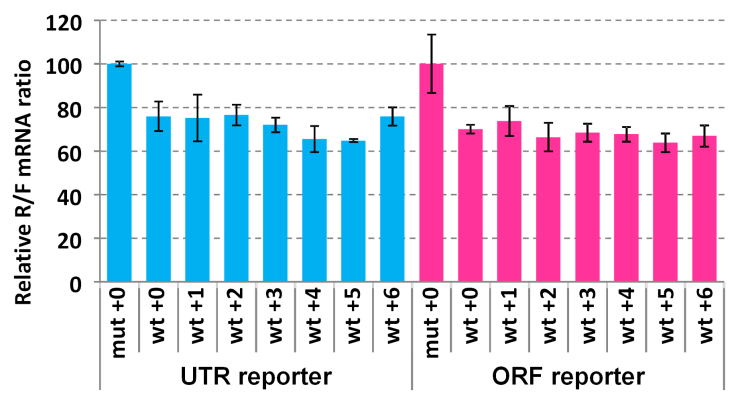
**

**Figure S2.** R/F mRNA ratios of reporter mRNAs relative to mRNA transcript with mutant G-rich sequence in MCF7 cells. The values are based on the ratio of mRNA transcripts of *Renilla* and firefly luciferases, which were evaluated by real-time PCR.


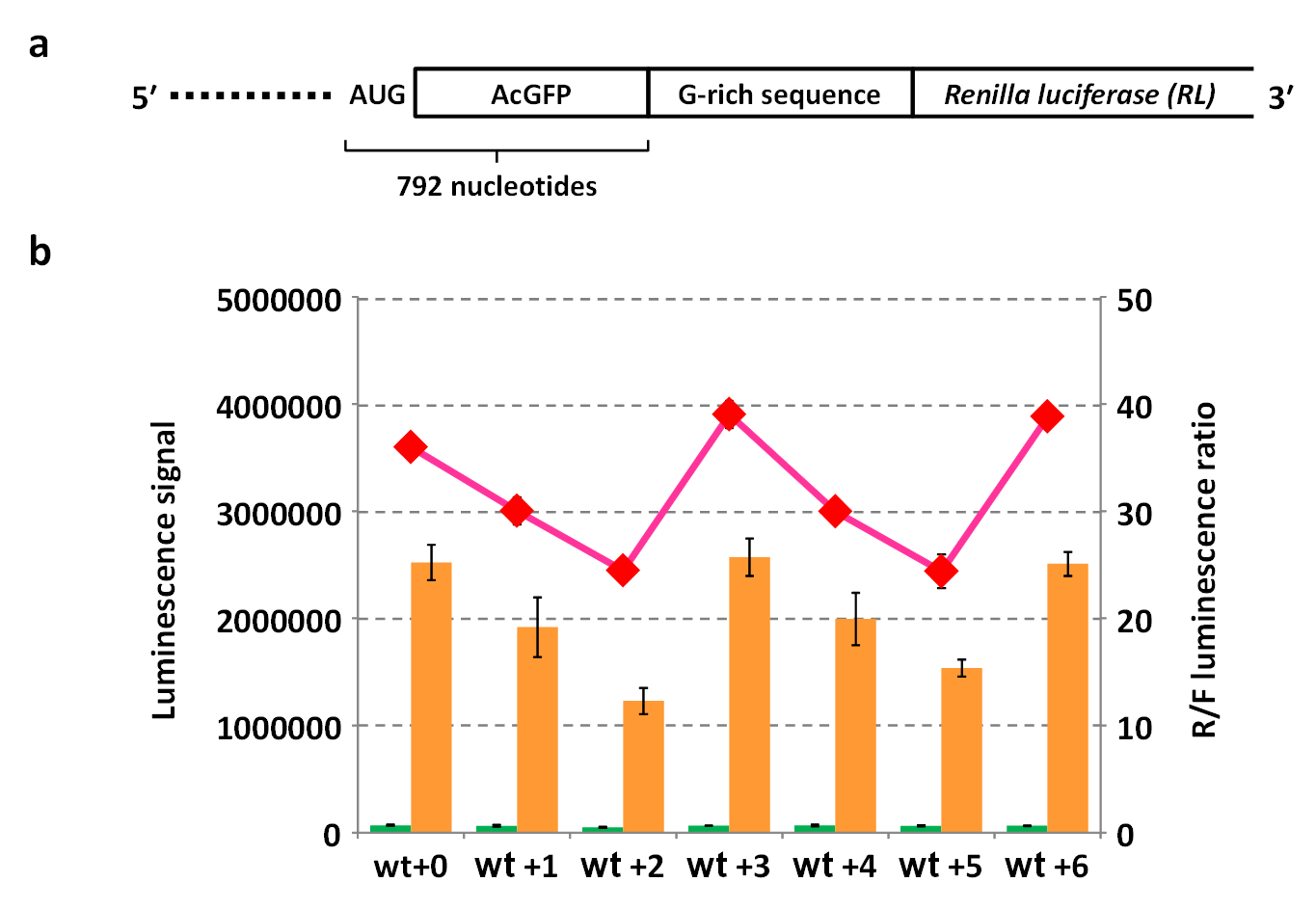


**Figure S3.** a) Design of reporter mRNA having G-rich sequence derived from *E. coli eutE* gene at 792-nucleotides downstream from start codon. Sequences of G-rich sequence variants inserted are shown in Figure 1b. b) Dual luciferase assay in cell lysates from MCF7 cells transfected with AcGFP-encoding reporter and control plasmids. Luminescence signals of firefly (green) and Renilla (orange) luciferases (left axes) are plotted in bar graphs; R/F luminescence ratios (red) (right axes) are plotted as data points. Values are means ± S.D. obtained from 5 wells.


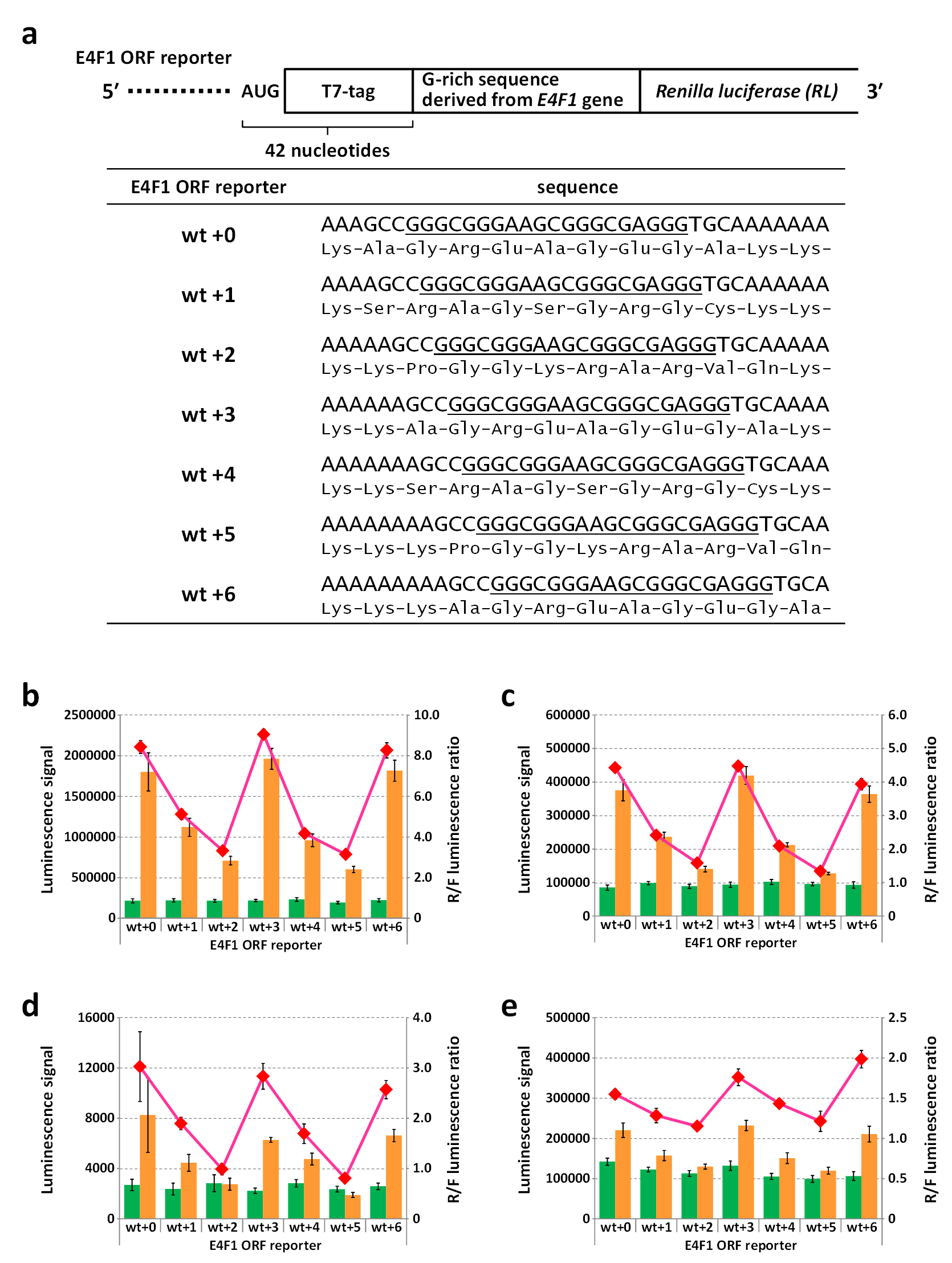


**Figure S4.** a) Design of reporter mRNA having G-rich sequence derived from human *E4F1* gene. Sequences of G-rich sequence variants are shown. G-rich regions are underlined. Amino acid sequences encoded by the G-rich sequences are shown under the nucleotide sequences. b-e) Dual luciferase assay in cell lysates from (b) MCF7, (c) Flp-Iin 293, (d) HeLa, and (e) HepG2 cells transfected with E4F1 ORF reporter and control plasmids. Luminescence signals of firefly (green) and Renilla (orange) luciferases (left axes) are plotted in bar graphs; R/F luminescence ratios (red) (right axes) are plotted as data points. Values are means ± S.D. obtained from 5 wells.
